# A Deep Learning Approach for Foot Trajectory Estimation in Gait Analysis Using Inertial Sensors

**DOI:** 10.3390/s21227517

**Published:** 2021-11-12

**Authors:** Vânia Guimarães, Inês Sousa, Miguel Velhote Correia

**Affiliations:** 1 Fraunhofer Portugal AICOS, 4200-135 Porto, Portugal; ines.sousa@fraunhofer.pt; 2 Faculty of Engineering, University of Porto, 4200-465 Porto, Portugal; mcorreia@fe.up.pt; 3 INESC TEC (Institute for Systems and Computer Engineering, Technology and Science), 4200-465 Porto, Portugal

**Keywords:** inertial sensors, gait analysis, foot trajectory, deep learning, long short-term memory (LSTM) networks

## Abstract

Gait performance is an important marker of motor and cognitive decline in older adults. An instrumented gait analysis resorting to inertial sensors allows the complete evaluation of spatiotemporal gait parameters, offering an alternative to laboratory-based assessments. To estimate gait parameters, foot trajectories are typically obtained by integrating acceleration two times. However, to deal with cumulative integration errors, additional error handling strategies are required. In this study, we propose an alternative approach based on a deep recurrent neural network to estimate heel and toe trajectories. We propose a coordinate frame transformation for stride trajectories that eliminates the dependency from previous strides and external inputs. Predicted trajectories are used to estimate an extensive set of spatiotemporal gait parameters. We evaluate the results in a dataset comprising foot-worn inertial sensor data acquired from a group of young adults, using an optical motion capture system as a reference. Heel and toe trajectories are predicted with low errors, in line with reference trajectories. A good agreement is also achieved between the reference and estimated gait parameters, in particular when turning strides are excluded from the analysis. The performance of the method is shown to be robust to imperfect sensor-foot alignment conditions.

## 1. Introduction

Gait performance is an important marker of mobility [1] and a predictor of cognitive decline [2] and mortality [3] in older adults. Mobility limitations and cognitive decline are highly prevalent with increasing age, and, in many cases, lead to negative outcomes such as hospitalization and falls [4,5]. An instrumented gait analysis allows the measurement of spatiotemporal gait parameters that can be used to assess motor and cognitive disabilities in older adults [4,6,7,8].

Optical motion capture systems are widely used in clinical research, and are currently considered the gold standard for the instrumented gait analysis [9]. Optical markers placed on the heel and toe allow the measurement of heel and toe trajectories, which, in turn, allow the extraction of gait events [10] and the measurement of relevant spatiotemporal gait parameters [11,12,13]. Optical motion capture systems are very expensive and time-consuming, and require specialized operators, which restricts their use to the laboratory setting [9]. Many studies propose the use of inertial sensors as an alternative to gold standard solutions [14]. Inertial sensors are cheaper and portable, offering an interesting alternative for the assessment of gait in clinical settings or in daily life [9,15].

Inertial sensors placed on the feet allow the complete evaluation of spatiotemporal gait parameters, being currently accepted as one of the best positions to evaluate gait [14]. Parameters such as the stride length, gait speed, cadence, swing width, and foot clearance, are important to assess walking performance in older adults [8] and can be obtained from this position [11,13,16,17]. Gait speed is considered one of the most relevant metrics to assess health-related outcomes in old age [2]. Foot clearance and, in particular, minimum toe clearance, have been connected to an increased risk of falling in older adults [18].

To evaluate spatiotemporal gait parameters, the foot trajectory is typically obtained by integrating acceleration two times [13,19]. This process requires not only a good estimation of sensor orientation (to isolate linear acceleration from the acceleration due to gravity), but also a good strategy to deal with cumulative double integration errors. To this purpose, techniques based on a zero-velocity drift correction (applied to the moments when the foot is in contact with the ground) are commonly employed [11,16,17,19]. The complexity of the required error handling strategies—often designed and optimized for a specific patient group [11,16,17,19]—still make the estimation of foot trajectories a challenging task.

Using some specific trigonometric relationships, previous studies could estimate the vertical trajectories of the heel and toe from inertial sensor data acquired at the dorsum of the foot [20,21]. In [20], shoe size was also used as an input, while in [21], heel and toe clearance metrics were obtained without previous knowledge of shoe dimensions. The estimation of heel and toe clearance metrics depends on the reliable estimation of foot trajectories [20,21]. Moreover, both studies assumed a rigid shoe model, which may not be realistic and introduce additional errors, e.g., due to the bending of the shoe at foot off [21].

To overcome the limitations of the conventional gait analysis and trajectory estimation methods, we propose a deep learning approach. We cast the gait analysis problem as a sequential learning problem that allows us to estimate heel and toe trajectories, while walking, on a stride-by-stride basis, using information from inertial sensors placed on the shoes. Deep learning has previously been applied to the context of gait analysis, but, to the best of our knowledge, this is the first study using deep learning techniques to estimate three dimensional (3D) heel and toe trajectories from inertial sensor data; therefore, emulating the trajectories’ data obtained from heel and toe markers in gold standard optical motion capture systems. Using these techniques, we overpass the need of designing and optimizing conventional approaches based on orientation estimation and double integration. Moreover, heel and toe trajectories are estimated in 3D, overcoming the limitations of previous heel and toe clearance estimation methods that could only evaluate the trajectories of the vertical component [20,21]. From heel and toe trajectories, an extensive set of spatiotemporal gait parameters can be obtained [10,11,12,13].

Previous works in gait analysis used deep learning to regress gait parameters directly from inertial sensor data. In [22], the stride length was obtained from inertial sensor data using a deep convolutional neural network. In [23], a similar architecture was used to estimate several parameters of interest, including the stride length, width, foot angle, heel contact time, and toe contact time. Even though these studies offered promising results, the proposed inference models could only predict gait parameters that were included in the training stage [22,23]. To integrate additional parameters, further training (and possibly a change in network architecture) would be required, which constitutes a limitation of these approaches. In contrast, the prediction of heel and toe trajectories allows the estimation of many spatiotemporal gait parameters of interest, which constitutes a more powerful approach.

Previous studies in odometry proposed the use of deep learning strategies to estimate the trajectories of a moving body. In the context of odometry, (naive) approaches based on double integration usually lead to critical errors due to integration drift, as zero-velocity updates are not always possible [24,25]. Studies have used deep neural networks to predict changes in pose, allowing the reconstruction of trajectories in 2D or in 3D [24,25]. In [26], and in [27], a deep neural network could estimate changes in distance and/or heading, enough to reconstruct the trajectories of a pedestrian. By predicting changes in pose, or in distance and heading, these studies rely on trajectory reconstruction mechanisms (incrementally composed) that, over time, would accumulate errors [24,25,27]. This formulation is, as such, inadequate for the context of a gait analysis.

To eliminate the dependency from previous strides and from external information (e.g., shoe length), we propose a new representation for heel and toe trajectories that is obtained for each stride. This formulation avoids the accumulation of errors due to trajectory reconstruction mechanisms, that, under this formulation, are not required. We propose, optimize, and evaluate a deep recurrent neural network to predict heel and toe trajectories from inertial sensor data, and use the predicted trajectories to extract and evaluate spatiotemporal gait parameters. We evaluate the results in a dataset comprising inertial sensor and reference data acquired from a group of young adults. We compare the results obtained using the deep learning approach with a conventional approach relying on orientation estimation and double integration techniques. Finally, we demonstrate that our model can generalize, being robust to different orientations of the sensor on the feet. This feature is important to ensure that the method is robust to imperfect sensor–foot alignment conditions, which may support its application in real scenarios outside the lab.

This work describes and discusses our findings in relation to previous works in related fields.

## 2. Materials and Methods

### 2.1. Dataset

**Figure 1:**
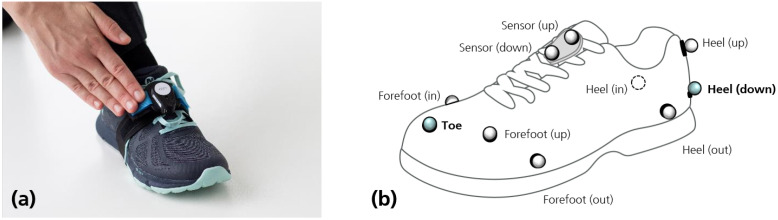
Placement of the inertial sensor (**a**) and markers (**b**) on the shoes.

The dataset thoroughly described in [28] was used in this study. It consisted of acceleration and angular rate—measured by two inertial measurement units (IMUs)—, and marker trajectories acquired by an optical motion capture system (Vicon, Oxford Metrics). IMU sensors were placed on the dorsum of the shoes, as shown in Figure 1a, tightly secured with an elastic band to reduce motion artifacts. Optical markers were placed on the shoes, using the marker set illustrated in Figure 1b, which included one marker near the heel and another marker near the toe (highlighted in Figure 1b). Markers’ trajectories were captured by 10 infrared cameras (Vicon Vero v2.2), resulting in a capture volume of around 3 per 8 m. Data were acquired at a sampling rate of 100 Hz, the rate most commonly used in gait analysis [14].

The dataset comprised walking data from 26 healthy young adults (average age of 29.2±5.3 years, 13 females and 13 males), each using their own shoes. Participants performed repeated walking trials under different conditions, including different speeds (comfortable, slower, and faster self-selected speeds) and different directions (clockwise and counterclockwise). Each walking trial included three consecutive laps over the lab’s capture volume, each lap comprising straight walking (along the length of the capture volume) and turns (along the width of the capture volume). Self-selected speeds ranged from 0.41 to 2.01 m/s (average 1.09±0.31 m/s).

The study received approval by the Ethical Committee of the University of Porto (81/CEUP/2019) and all participants provided written informed consent.

### 2.2. Reference Trajectories and Gait Parameters

Marker trajectories were used to detect gait events and parameters. Before detecting them, we estimated the steady periods of walking, i.e., the moments when the foot was not moving and was in contact with the ground. These periods were identified using a threshold-based approach—applied to the velocity of the centroid of heel and toe trajectories—, as described in [10]. These periods were used to estimate the vector normal to the horizontal plane of movement—resorting to Principal Component Analysis (PCA)—, which was used to calibrate the lab’s horizontal plane, as described in [28].

Reference gait events and parameters were automatically extracted from marker trajectories using a Python routine. Foot contact (FC) and foot off (FO) events were detected from local minima and maxima occurring on vertical heel velocity and vertical toe acceleration, using the method described in [10]. Before detecting gait events, trajectories were low pass filtered using a zero-lag bidirectional first order Butterworth filter (cutoff of 20 Hz) [28].

**Figure 2:**
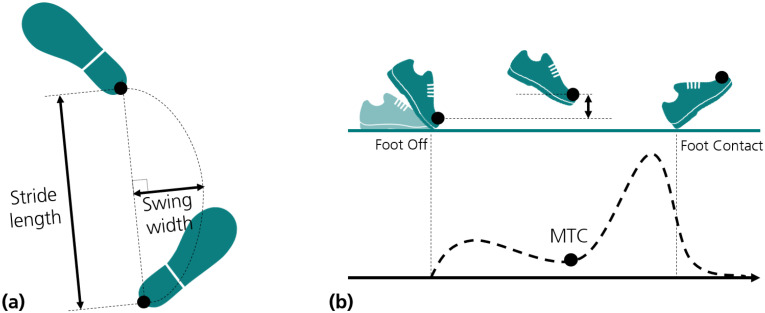
Definition of spatial gait parameters based on heel and toe trajectories: (**a**) Stride length and swing width. (**b**) Minimum toe clearance (MTC).

Temporal gait parameters—stride, stance, and swing duration—were obtained from FC and FO events. Cadence was calculated as the inverse of stride duration, in units of steps per minute [28]. Spatial gait parameters—stride length (SL), swing width (SW), and minimum toe clearance (MTC)—were calculated as illustrated in Figure 2, using the trajectories of the heel and toe. Gait speed was obtained by dividing stride length by its corresponding stride duration [28].

### 2.3. Data Preparation

Inertial sensor data were synchronized with reference trajectories using the maximum cross-correlation between acceleration magnitude—obtained from the IMU—and the centroid of heel and toe trajectories, as described in [28].

Before feeding data to the neural network, a series of preprocessing steps were conducted, which included segmentation of continuous recordings, coordinate transformations, padding to a fixed length, and normalization. Preprocessing steps were applied to right and left feet data without any distinction.

Spatial gait parameters, such as stride length, are typically defined within mid-stance events, i.e., between the midpoints of steady periods. For this reason, mid-stance events (detected from the reference trajectories) were used to segment the data. In [22], the best results for stride length estimation using a neural network were achieved when strides were defined between mid-stance events. Using this formulation, the start and end points of each segment (or stride) corresponded to instants when the foot was completely in contact with the ground, providing a stable basis for spatial metrics calculation and coordinates transformation—as described next.

**Figure 3:**
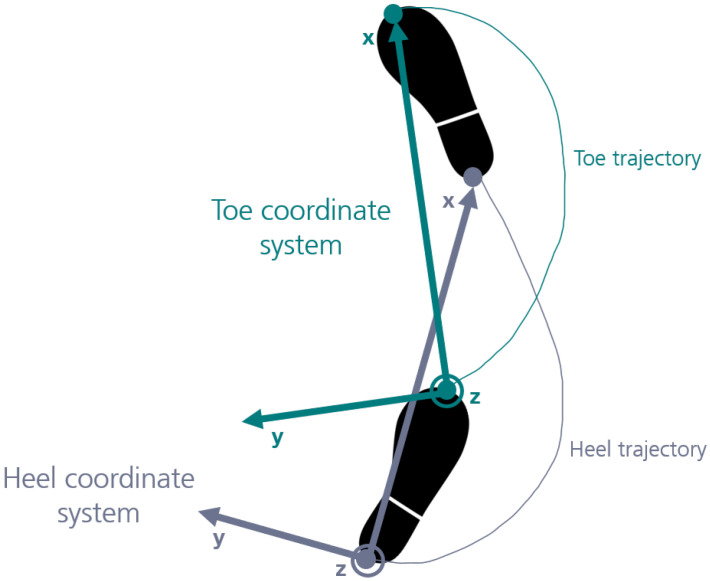
Coordinate systems of the heel and toe stride trajectories.

Considering that states such as attitude or position cannot be directly observed from inertial sensor data, they need to be derived from previous states and, for this reason, data segments are not independent [25]. To deal with these constraints, some authors proposed to estimate a change in state (delta) rather than the state of the system [24,25]. In this work, we proposed to represent trajectories within a stride using a new coordinate system that did not depend on the previous stride. The new coordinate system was obtained after subtracting the origin, followed by rotating the global coordinate system (lab) so that the main movement direction (formed by the vector connecting the start and end points of the trajectory in the stride, in the horizontal plane) corresponded to the x-axis in the new coordinate system. The vertical direction aligned with lab’s vertical direction (z-axis), and the y-axis was perpendicular to x and y axes. A new coordinate system was obtained for each stride, and for heel and toe trajectories independently, as illustrated in Figure 3.

The trajectories in this new coordinate system can be obtained from inertial sensor data resorting to double integration and orientation estimation techniques, without considering any inputs from previous strides [28]. While gravity provides a reference to the vertical component of trajectories (z-axis), horizontal components can be obtained (by double integration) using any convention (i.e., pointing to any horizontal direction), and latter transformed so that the x-axis can be derived from the vector connecting the start and end points of the trajectory in the stride [28]. This formulation ensures that strides are independent. Moreover, as heel and toe coordinate systems were independently defined, no prior knowledge concerning the relationship between heel and toe markers (e.g., their distance) was required under this formulation. Previous studies demonstrate that vertical heel and toe trajectories can be obtained from inertial sensor data acquired at the dorsum of the foot, without any additional input [21,28], which supports the theoretical background of the proposed deep learning approach. Gait parameters could be directly extracted from heel and toe trajectories under this new formulation, as explained in detail in Section 2.7.

Rotation was applied to inertial sensor data sequences for data augmentation purposes. Rotations were randomly sampled from a uniform distribution of quaternions, as proposed in [29]. Five randomly sampled artificial rotations were added to each data segment, so that different rotations were applied to each data segment and the number of sequences was increased sixfold. Generated rotations were uniformly distributed across the whole 3D space. With this process, we not only increased the total number of sequences in the dataset, but also introduced variations that may support a better generalization performance of the model, robust to different sensor orientations or sensor placement errors. Data augmentation through rotation is a common strategy applied in this domain [30,31,32].

To ensure that all sequences (inertial sensor data and trajectories) had the same size, they were padded with their initial value to obtain a fixed size of 256 samples—as proposed in [22].

The dataset was then split into three distinct partitions: training, validation, and test. Approximately 20% of the strides, from unique randomly selected participants, were included in the test set. The remaining 80% (60% for training, and 20% for validation—also from unique randomly selected participants) were used for optimization of hyperparameters and model fitting (Table 1).

Accelerations, angular rates, and trajectories were normalized using their respective maximum values, computed along all strides in the training set.

**Figure 4:**
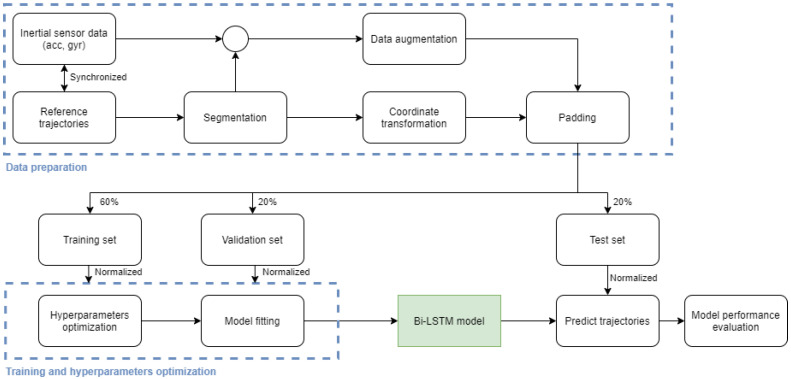
Data analysis process diagram: data preparation, network training, and model performance evaluation.

The operations concerning data preparation, network training, and model performance evaluation are summarized in Figure 4. Network training and model performance evaluation are presented in detail in the next sections.

### 2.4. Network Architecture

A stacked bidirectional long short-term memory (LSTM) recurrent network was used in this study, comprised of two bidirectional LSTM layers. LSTM networks are an extension of the vanilla recurrent neural network (RNN) architecture, designed to explore temporal dependencies within sequential data. The LSTM cell has a more complex structure and was shown to better handle long-term dependencies within the data [33]. LSTM networks have been proposed to solve problems dealing with wearable inertial sensor data, e.g., in the area of odometry [24,25], pedestrian dead reckoning [26], kinematics [15,31,34,35], attitude estimation [32,36], fall risk assessment [30], activity recognition [37,38], among others.

We specifically used LSTM layers in a bidirectional architecture, i.e., the network included forward and backward LSTM layers that were simultaneously trained, so that both time directions (past and future) were considered and contributed to the outcome [33]. The bidirectional architecture improved model performance in problems dealing with inertial sensor data, e.g., in [25], or in [39]. In the gait analysis domain, double integration with linear dedrifting or direct and reverse integration are typically applied to bound integration errors, considering that the foot should have a velocity of zero in the initial and final instants of the stride [19,40,41]. A bidirectional architecture was proposed here to deal with these domain-specific constraints. The outputs of forward and backward layers were combined using the sum.

The first bidirectional LSTM layer received the input sequences, i.e., acceleration and angular rate data, with dimension ($N_{strides}$× 256 × 6), where $N_{strides}$ corresponds to the number of strides, 256 is the length of the sequence, and 6 is the number of axes (3 axes for acceleration data plus 3 axes for angular rate). The second bidirectional LSTM layer output heel and toe trajectories arranged in a multidimensional sequence with the same dimension ($N_{strides}$× 256 × 6), where 6 represents the number of axes (3 axes for toe trajectories plus 3 axes for heel trajectories). Right and left inertial sensor data were fed to the network without any distinction.

### 2.5. Training and Hyperparameters Optimization

The neural network was trained to model the relation between inertial sensor data and heel and toe trajectories within a single stride. As a loss function, we used the mean squared error (*MSE*), as depicted in (1).

(1) $$MSE=\frac{1}{N}\sum_{n=1}^{T}{\left(y_{n}-{\hat{y}}_{n}\right)}^{2}$$

where $y_{n}$ and ${\hat{y}}_{n}$ are the flattened reference and estimated sequences, and *N* is the number of elements in a sequence (i.e., 256 samples multiplied by 6 axes).

For the training, we used the Adam optimizer, one of the most popular gradient-based optimization methods for stochastic learning [42]. Random subsets of the training set (called mini-batches) were shown to the optimizer in one iteration of the training loop (or epoch) to speed up the learning phase (stochastic learning). The data were re-shuffled at every epoch, to avoid bias errors due to training data order. The mini-batch error was summarized with the average of the individual MSE calculated for each sequence in the batch.

To achieve the best results, we optimized hyperparameters using an automated hyperband search [43], as provided by Keras-tuner [44]. In this process, we also explored the addition of a dropout layer after the first bidirectional LSTM layer, that would randomly drop a given number of nodes during training to prevent overfitting [45].

Hyperparameters comprised the number of units in the first bidirectional LSTM layer, the dropout rate (where a zero rate implied no dropout), the learning rate in Adam optimizer, and the size of the mini-batches. The configuration space considered in the hyperband search is shown in Table 2.

**Figure 5:**
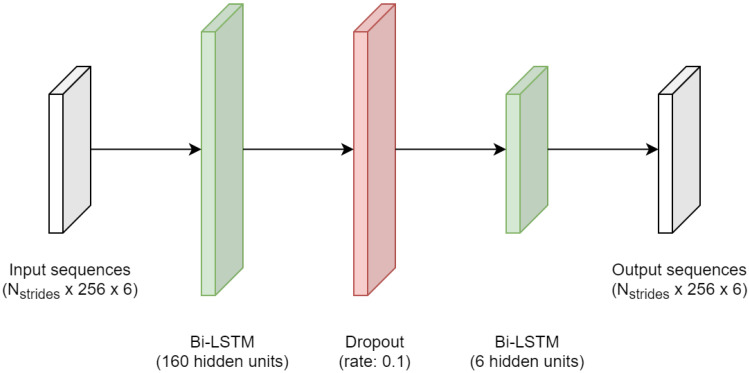
Neural network architecture.

The best hyperparameters were selected with basis on the validation loss, obtained from the sequences in the validation set. The hyperband was configured to run for a maximum of 350 epochs (expectedly higher enough to converge—according to some previous tests), using a reduction factor of three, and two hyperband iterations [43,44]. We also configured the hyperband search to perform three executions per trial—and summarize the trial with the average validation loss—to account for random influences (e.g., due to dropout, training batch selection, etc.). An early stop criterion was applied to the training process, so that the training would stop if no improvement in the validation loss was verified during the next 50 epochs. The optimal configuration obtained using the hyperband search is shown in Table 2, and the final network architecture is shown in Figure 5.

The best set of hyperparameters was used to train the final model, using the training set, a maximum number of 350 epochs, and early stopping. The model with best validation loss throughout training was chosen as the final one for the testing.

The networks were trained using Python (https://www.python.org/, accessed on 7 July 2021) 3.7.9, Tensorflow (https://www.tensorflow.org/, accessed on 7 July 2021) 2.2.0 and Keras (https://keras.io/, accessed on 7 July 2021) 2.3.0, using the NVIDIA^®^ V100 GPU with 16GB of memory.

### 2.6. Model Performance Evaluation

While the validation set was used to evaluate models during hyperparameters tuning and model fitting, the test set was used to determine the generalization performance of the model.

Because of the normalization applied during data preparation (Section 2.3), the model predictions (heel and toe trajectories) obtained from the test samples needed to be rescaled back to the original (physical) units, using the inverse operation, i.e., using the scaling parameters calculated from the training set. Contrarily to the training process, during testing, the full architecture of the network was used without dropping any connections.

To report the performance of the model we used MSE, mean absolute error (MAE), root mean squared error (RMSE) and average Euclidean distance. These metrics have been commonly used in the literature to report the performance of estimated trajectories [24,25,27].

### 2.7. Gait Parameters from Predictions

**Figure 6:**
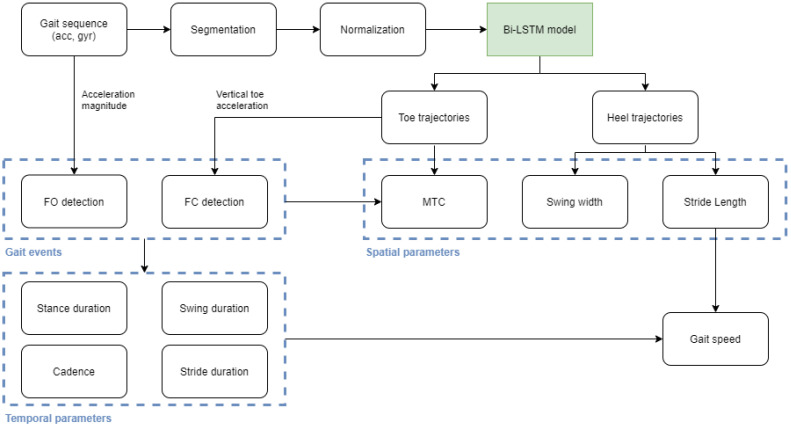
Data analysis process diagram: estimating gait parameters from predicted heel and toe trajectories.

Gait parameters were extracted from heel and toe trajectories, which, in turn, were obtained from inertial sensor data using the deep learning model. The full pipeline required inertial sensor data to be segmented, and each segment normalized with regard to sequence length and scale (using the scaling parameters calculated from the training set) before generating model predictions. The full data analysis process concerning the estimation of gait parameters from deep learning predictions is illustrated in Figure 6.

To segment inertial sensor data, steady periods were first detected using the angular rate energy detector [46]. A sliding window with 0.15 s was used to calculate the energy of the angular rate magnitude, as in [28]. The threshold was calculated with basis on the average of the energy, as described in [28]. Mid-stance events were used to segment continuous inertial sensor data recordings.

Generated segments were then padded with their initial values to obtain a fixed size of 256 samples. Using the scaling parameters calculated from the training set, segments were then normalized and used as an input to the neural network. Predicted trajectories were converted back to the original units using the scaling parameters of the training set, and expressed in meters.

**Figure 7:**
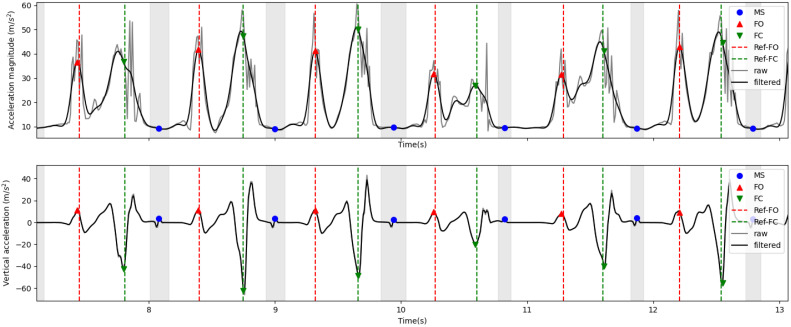
Events detection from acceleration magnitude (obtained from the inertial sensor data) and vertical acceleration (obtained from predicted toe trajectories). FO—foot off; FC—foot contact; MS—mid-stance; Ref-FO—reference FO; Ref-FC—reference FC.

FO and FC events were detected as described in [28]. The FO event was considered the first maximum after a steady interval, obtained from the acceleration magnitude (calculated from raw inertial sensor data). The FC event was considered an absolute minimum between FO and the next steady interval, obtained from the vertical acceleration. To obtain this signal, predicted toe trajectories were derived two times, revealing a signal pattern similar to the one used in [28] to extract FC events (in [28] vertical acceleration was calculated from inertial sensor data after the estimation of sensor orientation). Detected events and corresponding signals are shown in Figure 7.

Temporal gait parameters, i.e., stride, swing, and stance duration, were determined from FO and FC events. To estimate stride length, we calculated the maximum displacement achieved in the main direction of movement, i.e., the x-axis, in predicted heel trajectories. Swing width was directly obtained from the y component of predicted heel trajectories, considered the maximum absolute value obtained in this direction. MTC was estimated directly from the vertical component (z-axis) of the toe trajectories; it was obtained from a minimum between FO and the next FC event, as illustrated in Figure 2. Finally, gait speed was obtained by dividing stride length by its corresponding stride duration.

### 2.8. Instrument Comparison and Validation

This section describes the metrics used to compare gait parameters extracted from predicted trajectories (obtained from the inertial sensor data) and reference gait parameters (obtained from reference trajectories). All evaluation metrics were calculated using the test set.

FC events, and their corresponding stride gait parameters, were classified as true positive cases if they occurred within a tolerance of 0.1 s relative to the reference [28]. Only these cases were considered for comparison with the reference system. Reference strides without any corresponding IMU-derived candidate were classified as not detected.

IMU-derived and reference gait parameters were compared on a stride-by-stride basis, using the metrics of accuracy (mean of relative and absolute differences), precision (standard deviation of relative and absolute differences), and RMSE. We also reported the 95% limits of agreement, as introduced by Bland Altman [47]. The correlation between instruments was calculated using Pearson’s ($r_{p}$)—in case of normal distribution—or Spearman’s ($r_{s}$)—when data could not be assumed to be normally distributed. We also reported equivalence tests using an equivalence zone of ±5% of the average of the metric. Equivalence tests were based on paired *T*-test (*T*)—for parametric—or Wilcoxon signed-rank test (*W*)—for non-parametric. To choose the appropriate test, data were first tested for normal distribution using Shapiro–Wilk test.

As straight walking tests are most commonly used to assess gait disorders [7], we repeated the analysis considering the scenario where only straight walking strides were included. As in [11], turning strides were defined as strides with a turning angle above 20 degrees.

Finally, to test if network’s inference capabilities (as well as the resulting gait parameters) were robust to differences in sensor orientation, we applied the same method as described in [28]. To that purpose, we simulated multiple rotations of the IMU on the shoes. We sampled quaternions from a uniform distribution—uniformly distributed across all 3D space [29]—and used each generated quaternion to synthetically rotate raw inertial sensor data in a walking trial. We compared gait metrics extracted from the original sensor orientation with those extracted from a rotated version of the sensor. To quantify differences, we reported the Root Mean Square Deviation (RMSD), correlation, and equivalence tests, as described above.

A significance level (*p*-value) of 5% was used to evaluate results.

### 2.9. Comparison with the Conventional Gait Analysis Approach

The conventional gait analysis approach relies on orientation estimation and double integration methods, from which an estimate of 3D sensor trajectories can be obtained [11,13,16,17,19]. To obtain orientation relative to a fixed frame of reference, we integrated gyroscope data, and used the moments when the foot was in contact with the ground to obtain an initial estimate of sensor inclination relative to the vertical (based on gravity measured by the accelerometer). This method was previously described and validated for foot-worn inertial sensors [11,16]. Linear acceleration (i.e., acceleration excluding gravity) was obtained from the calculated orientation quaternions. Double integration of linear acceleration, expressed in the fixed frame of reference, allowed the estimation of sensor trajectories. To bound the errors, linear dedrifting was applied between zero-velocity intervals, as described in [16,40]. Sensor trajectories were expressed on a stride-by-stride basis, using the method described in Section 2.3. Gait events were obtained as described in Section 2.7, i.e., using acceleration magnitude to detect FO events and vertical sensor acceleration to detect FC events [28]. Stride length and swing width were obtained from the estimated sensor trajectories. MTC was estimated assuming a rigid shoe model and using trigonometric relationships as described in [21,28].

Sensor trajectories, estimated using the conventional approach, were compared with reference sensor trajectories obtained from the centroid of the two markers placed on the sensor (see Figure 1). Trajectories and gait parameters were evaluated using the mean and standard deviation of the absolute differences.

## 3. Results

### 3.1. Network Training and Trajectories Estimation

The optimal results of the hyperparameter search are shown in Table 2. The optimal configuration used a batch size of 100 samples, a learning rate of $1\times 10^{-3}$, and a dropout rate of 10%. For the first bidirectional LSTM layer, the best configuration was achieved with 160 units. Two hyperparameters—number of units in the first layer and batch size—were optimally defined at the upper and lower boundaries of the search space. The hyperparameter optimization process took approximately 1 day and 6 h.

**Figure 8:**
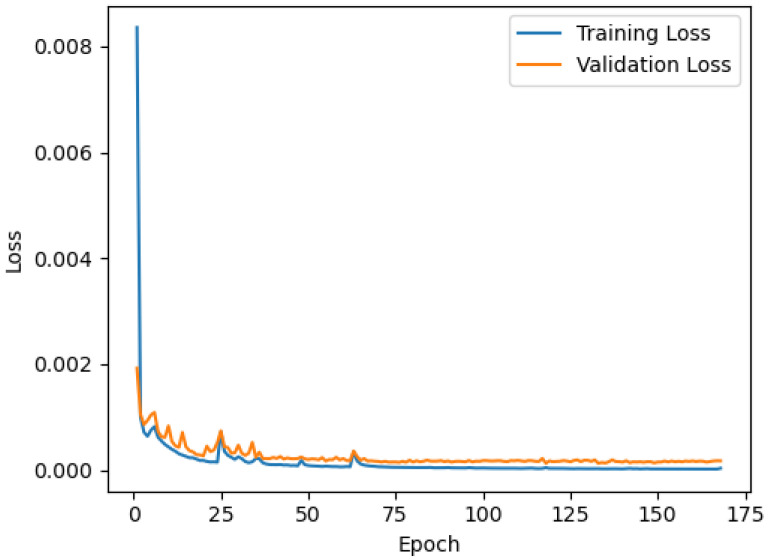
Mean squared error (loss) achieved while training the model with the best selection of hyperparameters. Loss was calculated from normalized trajectories.

Figure 8 shows the mean squared error (loss) achieved on the training and validation sets while training the model with the selected hyperparameters. The training loss decreased throughout the training, accompanied by a decrease in the validation loss. The best validation loss was achieved in epoch 118 due to early stopping; the training stopped after 50 epochs, at 168.

**Figure 9:**
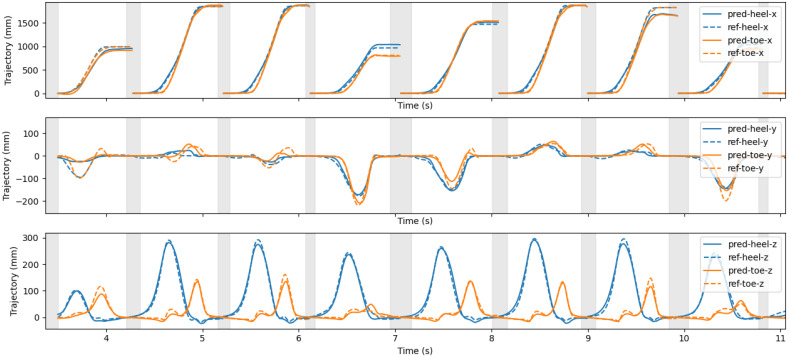
An example of predicted (full lines) and reference (dashed lines) trajectories, obtained from a sample in the test set. Heel and toe trajectories are shown in a different color.

The deep learning model was used to predict heel and toe trajectories from inertial sensor data. Figure 9 shows an example of predicted and reference trajectories, obtained from a sample in the test set. Each graph shows a different component of the trajectories: on the first graph, the x component is shown, representing the main movement direction (stride length); the second graph shows the y component, or, the width of the strides; the bottom graph shows the vertical component of the trajectories (z-axis), or heel and toe clearance.

In all the components (x, y, and z), the shapes of the predicted trajectories were generally in agreement with reference trajectories. Moreover, the temporal alignment of predicted sequences was in line with the reference (Figure 9).

The performance of the neural network is presented quantitatively in Table 3, using the metrics of MSE, MAE, RMSE, and the Euclidean distance. The performance on the validation set was higher than the one achieved on the test set for both heel and toe trajectories, for all metrics evaluated. Heel and toe trajectories were predicted with an equivalent performance both in validation and test partitions.

### 3.2. Gait Parameters Using the Deep Learning Approach

With basis on FC events (obtained from vertical acceleration derived from predicted toe trajectories), 2068 strides out of 2151 reference strides (i.e., approximately 96.1%) were considered true positive cases, i.e., only 83 strides (3.9%) were classified as not detected. FC events were detected with a relative error (i.e., relative difference between detected and annotated event times) of −0.01±0.02 s and an absolute error (i.e., absolute difference between detected and annotated event times) of 0.02±0.02 s. FO events (obtained from raw acceleration magnitude) were detected with a relative error of −0.01±0.03 s and an absolute error of 0.02±0.02 s. Thus, on average, FC and FO events were detected before the occurrence of the reference event.

The resulting performance on temporal and spatial gait parameters, obtained on the test set, is shown in Table 4.

Using the classification scheme proposed in [48], all metrics presented a high (between 0.70 and 0.90) or very high (above 0.90) correlation with the reference, except MTC which had a negligible correlation (below 0.30). The gait speed was estimated with a relative error of −2.2±10.5 cm/s and an absolute error of 5.8±9.0 cm/s. All metrics were practically equivalent (with p<0.01), except SW and MTC.

Table 5 summarizes the performance on the test set when turns were excluded from the analysis.

RMSE values improved for almost all metrics (except for stride and swing duration, where the RMSE value maintained) when turns were excluded from the analysis. Absolute errors (accuracy and precision) and relative errors (precision) also improved for most of the parameters. When turns were excluded, all metrics presented a very high (above 0.90) correlation, except SW—which had a high correlation (above 0.70)—and MTC—which maintained a negligible correlation (below 0.30). All metrics were considered practically equivalent (with p<0.01), except SW and MTC. Average relative and absolute errors of −2.2±7.0 cm/s and 3.9±6.2 cm/s were obtained for the gait speed.

### 3.3. Robustness to Changes in Orientation

Table 6 summarizes the results of the comparison between gait parameters extracted using original IMU data and simulated rotations of the sensor.

The correlation between variables was very high for all metrics (above 0.90), except for MTC, where the correlations were high (above 0.70). When data were synthetically rotated, all parameters remained practically equivalent, with p<0.01.

### 3.4. Comparison with the Conventional Gait Analysis Approach

Table 7 summarizes the comparison of the performance achieved with the deep learning approach and the conventional approach described in Section 2.9. As mentioned in Section 2.9, the conventional approach could only estimate 3D sensor trajectories, while the deep learning approach was able to predict 3D heel and toe trajectories. Mean absolute errors were lower for heel and toe trajectories—predicted using the deep learning model—than for sensor trajectories—estimated using the conventional approach—, whether turns were included on not. The resulting gait parameters were also estimated with lower mean absolute error and standard deviation when using the deep learning approach. The differences in performance were more apparent on spatial gait parameters—stride length, SW, and MTC—and gait speed than on temporal gait parameters. When turns were excluded from the analysis, the trajectories and gait parameters were estimated with a lower error. In both scenarios (i.e., including and excluding turns), better results were achieved when the deep learning approach was employed.

## 4. Discussion

In this study, we proposed a novel IMU-based gait analysis solution based on a deep recurrent neural network. The model was used to estimate foot trajectories, being proposed as an alternative to the traditional orientation estimation and double-integration approaches found in the literature [11,16,17,19,20,21,28]. A stacked bidirectional LSTM network was used to estimate heel and toe trajectories, from which clinically relevant gait parameters could be extracted. Dissimilar to the gold standard instrumented gait analysis conducted in the lab, the proposed method relies on the use of foot-worn wearable sensors that can be applied outside the lab, e.g., in a clinical setting. To better support operation under an imperfect sensor placement (with regard to its orientation on the shoes), we proposed a data augmentation scheme, and evaluated the robustness of the model to changes in orientation. Results were evaluated in a dataset comprising foot-worn inertial sensor data and reference data acquired from a group of young adults.

### 4.1. Problem Formulation

The network architecture used in this study was inspired by past works in related fields, namely, in [25], [34] or [35]. In kinematics, in particular, LSTM networks were proposed to predict multidimensional sequences of kinematic variables—e.g., joint angles and moments—from multidimensional sequences of inertial sensor data—acceleration and angular rotation [34,35]. Using a similar approach, we proposed to predict multidimensional trajectories from multidimensional IMU data. Final results were reported from data on the test set that included samples (and subjects) never seen by the model during the training process. These results, thus, represent the generalization performance of the model.

To optimize the results, hyperparameter selection (using the hyperband search) was conducted prior to model fitting, and the validation set was used to monitor training performance (Figure 8). The optimal results of the hyperparameter search, in particular, the two hyperparameters—number of units on the first LSTM layer and batch size—were equal to the upper or lower boundaries of the search space (Table 2). Although this may indicate that better configurations could have been obtained by searching among wider configuration spaces, due to training time constraints, the boundaries of the search were not further decreased or increased, as it would result in a significant increase in training time. Still, trajectories were predicted with a low error compared to the reference, already denoting satisfactory results and a good trade-off between speed and performance.

To avoid overfitting and a reduced generalization of the model, the training was stopped when the validation loss could not improve any further. The best validation loss was achieved before reaching the maximum number of epochs (i.e., before reaching 350 epochs), which confirmed the adequacy of this number. A dropout regularization was also introduced to reduce overfitting during training. The performance of the model during training (Figure 8) indicated that both measures were effective in preventing overfitting, resulting in learning curves (training and validation losses) with a similar pattern.

We proposed a novel representation for heel and toe trajectories to ensure that sequences were independent and did not rely on previous sequence information nor calibration procedures. The new formulation also represented heel and toe trajectories independently from each other; therefore, avoiding the need to input additional information, such as the relationship between them (e.g., distance and/or angle).

Previous studies from the areas of odometry [24,25] and pedestrian dead reckoning [27] approached the concern of obtaining inter-segment independence by proposing a network output that estimates a change in the state rather than the actual state of the system. The results were reported in terms of positioning errors of the reconstructed trajectories (e.g., [24] reports an RMSE of 48.2 cm, evaluated in excerpts with 20 s of a single person walking; [27] reports an average Euclidean distance of 1.47 m, evaluated in paths with an average length of 90.3 m). Considering that reconstructed trajectories were obtained from changes in the state (given an initial state of the system), estimated trajectory errors were cumulative [24,25], and, for this reason, these approaches were not suitable to our problem concerning the stride-by-stride gait analysis.

On the other hand, as we considered a formulation that was inter-segment independent, it was not possible for us to reconstruct the full trajectory of the walking trial, as the relationship between segments was missing from our predictions. This is not, however, a concern of the gait analysis, and should not be considered a limitation of the study. Rather, as errors did not accumulate along the stride nor stride to stride (as can be observed in Figure 9), errors affected each stride (and respective gait parameters) independently.

Our formulation was designed to ensure an end-to-end approach capable of predicting heel and toe trajectories from inertial sensor data, and, from the trajectories, extract clinically relevant gait parameters. However, the proposed formulation did not allow us to estimate turning angles from the trajectories, which constitutes a limitation of our approach. Although straight walking tests are more commonly employed to assess older adults’ gait performance [7], in specific patient populations, e.g., in patients with Parkinson’s Disease, turning deficits are very pronounced and should be part of the gait assessment [49]. To approach these specific patient populations, turning angles (and other specific biomechanical features from turning [49]) could be estimated separately, for instance, using another neural network, or, alternatively, integrated in the current neural network using a multi-task approach [50]. The estimation of the turning angles was considered out of the scope of the current research.

### 4.2. Deep Learning-Based Gait Analysis Performance

A good agreement between IMU-derived and reference gait parameters was achieved in our study. Temporal parameters, as well as the stride length and speed, were evaluated with high or very high correlations, and demonstrated equivalence with the reference method (Table 4 and Table 5).

The swing width and MTC presented the lowest agreement with reference parameters (as evidenced by correlation coefficients and equivalence tests). Both parameters presented a low average value, and the swing width, in particular, was further lowered when turns were excluded. In both metrics, small errors had a higher impact on performance due to the narrow range of the parameters. According to [51], the use of inertial sensors to estimate MTC remains a challenging task, due to this effect.

The performance of the estimated gait parameters was, in general, improved when turns were excluded from the analysis, which implied that evaluating turns constituted a more challenging task for the network. Turns may present a more complex pattern—stride trajectories, accelerations, and angular rates differ from straight walking—due to feet adaptations while turning [52], and were, as such, more difficult to evaluate. A similar observation was also determined by [28]; while this was true for almost all parameters, MTC seemed to have been less influenced by this effect, as errors remained at the same order. The average of the metric lowered when turns were excluded, but the differences were very small, which may imply little effect of turning on MTC in our results. Previous studies found significant differences in foot clearance while walking straight and turning [16,53], which contradicts our results. The detection of temporal parameters (i.e., gait events) seemed to be robust to the variability in strides due to turning. Possibly, optimizing and training a deep neural network that would only include straight walking strides could optimize the results for straight, steady walking; however, by including less variability on the data, also the generalization of the method could possibly drop. Results showed that the proposed method may be adequate to evaluate straight walking tests, with important applications in geriatrics and health-related areas.

Supported by our experiments reported in Table 6, the performance of our method seemed to not depend on the precise alignment of the sensors on the feet. The proposed data augmentation scheme allowed the model to capture the relationships between inertial sensor data and stride trajectories regardless the orientation of the sensor on the foot. Due to the intrinsic errors from the predictions, the resulting RMSD values in Table 6 were not equal to zero, although all metrics were considered practically equivalent. This is an important requirement for solutions designed to operate outside the lab, in real settings, where the careful alignment of the sensor on the foot may not be attained for practical reasons. This feature may potentially simplify the data acquisition process, while increasing trust in the context of the clinical gait analysis.

### 4.3. Comparison with the Conventional Gait Analysis Approach

A simple conventional gait analysis approach—resorting to orientation estimation and double integration—was implemented in this study for a comparison with the proposed deep learning-based gait analysis solution. The conventional approach resorts to the double integration of linear acceleration—expressed in a fixed frame of reference—obtained from raw acceleration after subtracting the gravity component. The orientation of the sensor relative to the vertical—obtained after integrating angular rate—allows the estimation of the gravity component during movement. As both methods depend mostly on integration (double integration of linear acceleration to obtain sensor trajectories and integration of angular rate to obtain sensor orientation), errors were cumulative along the stride [11,13,16,17,19]. Even though linear dedrifting was applied to mitigate some of the integration errors [16,40], the conventional approach revealed a worse performance than the deep learning approach, which was evidenced by the results presented in Table 7.

As sensor trajectories were determined with a higher error—using the conventional approach—than the heel and toe trajectories—obtained using the deep learning approach—, the resulting gait parameters were also obtained with a higher error. Spatial gait parameters, in particular, were affected by the errors in estimated foot trajectories, resulting in higher errors when the conventional approach was applied. Similarly to the deep learning-based approach, the performance of the metrics improved when turns were excluded from the analysis. As turns presented a more complex pattern, it is very likely that trajectory estimation errors were higher during the turns due to the cumulative behavior of the errors in the conventional approach.

While the proposed deep learning approach could estimate 3D heel and toe trajectories, the conventional approach estimated 3D sensor trajectories. To obtain MTC, trigonometric relationships—assuming a rigid shoe model—were employed by the conventional approach, which allowed the estimation of the vertical component of the toe trajectories [20,21]. The resulting values accumulated errors from the processes of estimating the sensor trajectory and estimating vertical toe trajectory. The proposed deep learning model was able to predict 3D toe trajectories directly from inertial sensor data, resulting in lower errors for the MTC estimation.

### 4.4. Framing our Method within the State-of-the-Art

To detect gait events, in our study, we used the method described in [28], proposed to ensure robustness to differences in sensor orientation on the feet [28]. Our study used the same dataset described in [28], while [28] estimated FC events from vertical acceleration—obtained after estimating sensor orientation—, we calculated vertical acceleration directly from predicted toe trajectories. Although the source of the signal to extract FC events differed from [28], the performance on gait event detection was very similar, which demonstrated the robustness of our method in predicting toe trajectories.

The resulting temporal metrics—stride, swing, and stance duration—had average absolute errors on the order of the 0.02 s (i.e., two samples at 100 Hz), with standard deviations ranging from 0.02 s to 0.04 s. These results were in line with the state-of-the-art [13,17]. Using a deep learning approach to directly regress gait parameters from inertial sensor data, [23] reports lower precision on the estimation of temporal parameters (±0.07 s for stride, ±0.05 s for swing, and ±0.07 for stance duration). Although we also relied on the outcomes of a neural network to detect FC events, the results achieved by our method demonstrated a more consistent performance.

The performance on the estimation of spatial gait parameters was also according to past works in the field, although, compared to some works, lower precision was achieved in some of the parameters. Using the same dataset, and although reporting results for a different set of subjects, [28] reports relative errors of −3.5±9.7 cm for the stride length and −3.1±9.2 cm/s for gait speed (i.e., less accuracy but better precision) using an orientation-invariant, double integration-based, approach. Better accuracy and precision (relative errors of 1.5±6.8 cm and 1.4±5.6 cm/s) were also obtained in [16] with a double-integration approach, using a dataset with young and elderly volunteers performing U-turns and eight-turns.

With a deep convolutional neural network, [22] reports a relative error of 0.01±5.37 cm for stride length. In [23], using a similar approach, stride length was estimated with a relative error of −0.15±6.09 cm. Although these results seemed more promising than the results we presented in this work, we need to consider that the dataset used in both studies referred to a group of geriatric inpatients walking straight. In our results, when we excluded turns from the analysis, the precision improved, resulting in −2.6±5.4 cm for the stride length and −2.2±7.0 cm/s for speed, which were already in line with previous works. Using a double integration approach, Ref. [17] reports relative errors of −0.3±8.4 cm (stride length) in a group of geriatric inpatients walking straight. In [13], mean relative errors of −5.4±3.1 cm and −3.4±3.9 cm/s (i.e., better precision) were obtained for stride length and gait speed in a group of young adults walking straight.

MTC, the parameter with the lowest performance in this study, achieved results on the level of previous works. In [20,21], a method based on double integration and a trigonometric relationship was applied to determine heel and toe trajectories from IMU data acquired at the dorsum of the feet. The method proposed by [20] required, additionally, the shoe size as an input. These studies reported a relative error of 1.3±0.9 cm [20] and 1.7±0.7 cm [21] for MTC. Our results were, thus, in line with the state-of-the-art, although—as previously discussed—errors remained very high compared to the low values of the parameter.

Most of these studies from the literature depend on the precise alignment of the sensors on the body, e.g., [13,16,17]. Moreover, by being conducted in laboratory settings, results reported by most of the studies were not capturing the imperfections of a poor sensor–foot alignment and, as such, may not represent the actual results that would be achieved in a more realistic setting. Deep regression models, as proposed by [22,23], also did not capture possible errors in sensor orientation, as the training set is limited to a single orientation. As discussed previously, our method relaxed this assumption by integrating adequate data augmentation techniques, which resulted in a gait analysis solution that was, in principle, robust to changes in sensor orientation. Nevertheless, additional tests may be required to confirm the robustness of our method in practical conditions.

As we could verify from the above discussion, deep learning models do not necessarily lead to better results compared to traditional approaches resorting to double integration (e.g., see [22] versus [13]). However, we should mention that most of the studies resorting to double integration developed their algorithms and reported their results using the same dataset [11,13,16,17,21], and, for this reason, they were not assessing the generalization performance of the method and may have been too optimistic. Contrarily, studies employing deep learning approaches are very careful with data splitting, so that part of the data is never seen by the model during training and can be used to assess its generalization performance [22,23]. Although double-integration and zero-velocity update methods rely on geometric and physical reasoning—and, for that reason, they are not so much dependent on the data—, this should be referred to as a possible limitation of these studies, whose results must be interpreted with caution.

More generally, results from the different studies should be compared and interpreted carefully, considering the different properties of the datasets employed. Different protocols (e.g., walking paths, walking speeds, sensors characteristics, etc.), subjects (e.g., young or older adults) and reference systems (e.g., optical motion capture systems or pressure sensitive walkways) may have an impact on the characteristics of the datasets that limit their comparability. Results are highly heterogeneous among the different studies; thus, a fair and robust comparison of performance is not always possible.

Compared to deep regression models [22,23] (proposed to extract gait metrics directly from inertial sensor data), our approach seemed to provide better precision for temporal metrics, and equivalent results for stride length. To regress gait parameters directly from inertial sensor data, deep regression models need to implicitly learn how to map foot trajectories from inertial sensor data. In our approach, this relation was addressed explicitly, which may constitute an advantage. On the other hand, the MSE loss used for training only captured a summary of the prediction performance, not focusing on the most important instants, or events, used to extract gait parameters later on. This may explain why our approach did not display a clear advantage concerning gait parameters estimation. As an additional advantage, our approach allowed not only the estimation of gait parameters, but also the evaluation of heel and toe trajectories on a stride-by-stride basis, e.g., resorting to the visualization and analysis of lateral profiles (as in [13,54]). Additionally, heel and toe trajectories enabled the extraction of additional gait parameters (e.g., heel clearance metrics) that, unless considered during the training of deep regression models, could not be extracted using these methods.

### 4.5. Limitations

Due to their black-box nature, deep learning models are often difficult to explain and, for that reason, they usually lack trust on how well they will generalize [55]. Dissimilar to more traditional gait analysis approaches found in the literature—that rely on geometric and physical reasoning—data-driven deep neural networks are implicitly dependent on the data used for training, which should, ideally, capture as much variability as possible, to allow a good generalization of the model [56].

This study assessed the performance of the deep learning model on a considerably high number of strides (after data augmentation); however, the number of participants included on training and test sets was relatively low. To better assess the generalization of the model, a cross-validation setup could have been applied, but, due to training time constraints, we opted for a single training-test split. Additional participants would need to be included to assess the generalization performance of the model in a more representative set. Additionally, only young adults were included in the study. Although the system was being designed envisioning the assessment of gait performance in older adults, this group was not included in this preliminary study, which constitutes a limitation.

As for the reference system, in this study, we used an optical motion capture system. Although it is currently considered the gold standard for a gait analysis, possible errors can also be introduced by the reference. Errors due to the wrong labeling of markers or inaccuracies on gap filling operations (which rely on manual operation) are common [57,58], and may lead to wrongly defined stride trajectories. The fact that many strides are included in the training set may mitigate the effects of training with inaccurate trajectories, yet, these effects should not be neglected.

## 5. Conclusions

This work proposed a novel approach for foot stride trajectory estimation in gait analysis. A deep recurrent neural network was proposed and optimized to allow the estimation of heel and toe trajectories on a stride-by-stride basis. To support this formulation, stride trajectories were represented in a new coordinate system that ensured inter-stride independence. Gait parameters were extracted from predicted trajectories and evaluated on a dataset comprising foot-worn inertial sensor data and reference walking data acquired from a group of young adults.

We demonstrated that bidirectional LSTM networks can be applied to the context of a gait analysis, in a sequence-to-sequence setup, to obtain heel and toe trajectories, using as an input the sequences from raw inertial sensor data. Our results showed that clinical gait parameters could be extracted from predicted stride trajectories, achieving an overall good agreement with the reference system. The proposed solution was also robust to imperfect sensor–foot alignment conditions, which may support its application in real scenarios.

Future work should investigate the performance of the method in additional datasets (with different persons) for a better evaluation of the generalization performance. It should also address specific groups, e.g., older adults with mobility limitations and/or cognitive impairment, for which the validity and robustness of the method should be assessed. The estimation of turning angles should be explored in future work to allow for a more detailed analysis of walking in specific patient populations. Additional gait parameters, e.g., heel clearance, may also be included. Future work should finally focus on demonstrating the clinical value of the proposed gait analysis solution.

Although there is certainly room for improving the proposed method, results achieved at the current stage of work are already encouraging. The prediction of foot trajectories from wearable solutions is highly relevant to assist clinical decision making in gait-related disorders, offering an interesting alternative for the assessment of gait in clinical settings or ambulatory conditions.

## Figures and Tables

**Table 1:** Dataset splitting.

Partition	Number of Subjects	Number of Strides
Training set (60%)	14	31,470
Validation set (20%)	6	13,872
Test set (20%)	6	12,474

**Table 2:** Hyperparameters optimization.

Parameter	Configuration Space	Optimal Configuration
Number of Units	32–160	160
Dropout	0–0.30	0.1
Learning Rate	$1\times 10^{-6}$–$1\times 10^{-2}$	$1\times 10^{-3}$
Batch Size	100–400	100

**Table 3:** Performance of the neural network on validation and test sets.

Metric	Validation Set		Test Set	
	Heel Traj.	Toe Traj.	Heel Traj.	Toe Traj.
MSE (mm^2^)	538.0	534.6	1043.7	1087.7
MAE (mm)	7.6	7.0	8.6	8.2
RMSE (mm)	14.2	13.3	17.2	16.8
Eucl. Dist. (mm)	17.9	16.6	20.2	19.5

**Table 4:** Performance on the test set, including turns (*n* = 2068 strides). Shown are mean values (standard deviation), limits of agreement, RMSE, correlation, and equivalence interval (*p*-value). ^†^ All correlations were based on Spearman and have p<0.01. ^‡^ Equivalence tests were based on Wilcoxon signed-rank test.

Parameter	IMU	VICON	Rel. Error	Abs. Error	Lim. Agr.	RMSE	Corr. ^†^	Equival. ^‡^
Stride dur. (s)	1.15(0.20)	1.15(0.20)	0.00(0.04)	0.02(0.04)	[−0.08,0.08]	0.04	0.99	±0.06(0.0)
Swing dur. (s)	0.40(0.07)	0.39(0.06)	0.00(0.04)	0.02(0.03)	[−0.07,0.08]	0.04	0.88	±0.02(0.0)
Stance dur. (s)	0.75(0.15)	0.76(0.16)	−0.01(0.03)	0.02(0.02)	[−0.06,0.05]	0.03	0.98	±0.04(0.0)
Cad. (st/min)	107.5(17.4)	107.5(17.8)	0.1(4.7)	1.8(4.4)	[−9.2,9.4]	4.7	0.99	±5.37(0.0)
SL (cm)	126.7(27.3)	129.4(28.4)	−2.6(10.0)	5.8(8.6)	[−22.3,17.0]	10.4	0.94	±6.47(0.0)
Speed (cm/s)	115.9(39.3)	118.0(40.0)	−2.2(10.5)	5.8(9.0)	[−22.7,18.4]	10.7	0.97	±5.90(0.0)
SW (cm)	9.4(10.3)	10.4(10.8)	−1.0(5.8)	2.6(5.3)	[−12.3,10.3]	5.9	0.88	±0.52(1.0)
MTC (cm)	1.7(0.4)	1.9(0.8)	−0.2(0.8)	0.6(0.6)	[−1.9,1.5]	0.9	0.20	±0.10(0.3)

**Table 5:** Performance on the test set, excluding turns (*n* = 1108 strides). Shown are mean values (standard deviation), limits of agreement, RMSE, correlation, and equivalence interval (*p*-value). ^†^ All correlations were based on Spearman and have p<0.05. ^‡^ Equivalence tests were based on Wilcoxon signed-rank test.

Parameter	IMU	VICON	Rel. Error	Abs. Error	Lim. Agr.	RMSE	Corr. ^†^	Equival. ^‡^
Stride dur. (s)	1.14(0.21)	1.15(0.21)	0.00(0.04)	0.01(0.04)	[−0.08,0.08]	0.04	0.99	±0.06(0.0)
Swing dur. (s)	0.39(0.07)	0.39(0.05)	0.00(0.04)	0.02(0.04)	[−0.08,0.08]	0.04	0.92	±0.02(0.0)
Stance dur. (s)	0.75(0.16)	0.76(0.16)	−0.01(0.02)	0.02(0.02)	[−0.05,0.04]	0.02	0.99	±0.04(0.0)
Cad. (st/min)	107.9(17.5)	107.7(17.4)	0.2(2.8)	1.3(2.5)	[−5.3,5.6]	2.8	0.99	±5.39(0.0)
SL (cm)	136.1(23.3)	138.8(24.6)	−2.6(5.4)	3.8(4.7)	[−13.3,8.0]	6.1	0.98	±6.94(0.0)
Speed (cm/s)	124.8(38.2)	127.1(39.3)	−2.2(7.0)	3.9(6.2)	[−15.9,11.4]	7.3	0.99	±6.35(0.0)
SW (cm)	2.7(2.2)	3.5(2.1)	−0.8(1.8)	1.3(1.5)	[−4.3,2.7]	2.0	0.76	±0.17(1.0)
MTC (cm)	1.6(0.3)	1.8(0.7)	−0.2(0.8)	0.5(0.6)	[−1.7,1.3]	0.8	0.08	±0.09(0.2)

**Table 6:** Random orientation simulation results (*n* = 2139 strides). Shown are mean values (standard deviation), RMSD, Correlation—with Spearman or Pearson—, and equivalence interval (*p*-value). ^†^ All correlations have *p* < 0.01. ^‡^ All equivalence tests are based on Wilcoxon signed-rank test.

Parameter	Original Orientation	Random Orientation	RMSD	Correlation ^†^	Equivalence ^‡^
Stride dur. (s)	1.17(0.27)	1.17(0.27)	0.03	$r_{s}=0.99$	±0.06(0.0)
Swing dur. (s)	0.40(0.07)	0.40(0.06)	0.03	$r_{s}=0.92$	±0.02(0.0)
Stance dur. (s)	0.77(0.23)	0.77(0.23)	0.02	$r_{s}=0.99$	±0.04(0.0)
Cad. (st/min)	106.4(18.5)	106.4(18.4)	2.5	$r_{s}=0.99$	±5.32(0.0)
SL (cm)	126.1(27.4)	126.8(26.3)	5.7	$r_{s}=0.98$	±6.34(0.0)
Speed (cm/s)	114.3(40.0)	114.7(38.5)	6.5	$r_{s}=0.99$	±5.73(0.0)
SW (cm)	9.3(10.3)	9.5(10.2)	2.3	$r_{s}=0.94$	±0.47(0.0)
MTC (cm)	1.7(0.4)	1.6(0.4)	0.3	$r_{s}=0.78$	±0.08(0.0)

**Table 7:** Comparison of the performance achieved with the deep learning approach and the conventional approach. Shown are mean absolute errors (standard deviation), reported for the samples in the test set.

Parameter	Including Turns		Excluding Turns	
	Deep Learning Approach	Conventional Approach	Deep Learning Approach	Conventional Approach
Trajectories (mm)	8.7(14.7) (heel) 8.7(13.2) (toe)	9.8(12.7) (sensor)	7.4(16.3) (heel) 7.5(14.8) (toe)	8.6(13.0) (sensor)
Stride dur. (s)	0.02(0.04)	0.02(0.05)	0.01(0.04)	0.02(0.05)
Swing dur. (s)	0.02(0.03)	0.03(0.04)	0.02(0.04)	0.02(0.05)
Stance dur. (s)	0.02(0.02)	0.02(0.02)	0.02(0.02)	0.02(0.02)
Cad. (st/min)	1.8(4.4)	1.9(4.8)	1.3(2.5)	1.4(2.9)
SL (cm)	5.8(8.6)	10.1(10.9)	3.8(4.7)	8.1(8.1)
Speed (cm/s)	5.8(9.0)	9.4(11.2)	3.9(6.2)	7.6(9.6)
SW (cm)	2.6(5.3)	3.5(6.2)	1.3(1.5)	1.9(4.2)
MTC (cm)	0.6(0.6)	1.5(1.0)	0.5(0.6)	1.4(0.8)

## Data Availability

Not applicable.
